# The Risk Instrument for Screening in the Community (RISC): a new instrument for predicting risk of adverse outcomes in community dwelling older adults

**DOI:** 10.1186/s12877-015-0095-z

**Published:** 2015-07-30

**Authors:** Rónán O’Caoimh, Yang Gao, Anton Svendrovski, Elizabeth Healy, Elizabeth O’Connell, Gabrielle O’Keeffe, Una Cronin, Estera Igras, Eileen O’Herlihy, Carol Fitzgerald, Elizabeth Weathers, Patricia Leahy-Warren, Nicola Cornally, D. William Molloy

**Affiliations:** Centre for Gerontology and Rehabilitation, University College Cork, St Finbarrs Hospital, Douglas Rd, CorkCity, Ireland; UZIK Consulting Inc., 86 Gerrard St E, Unit 12D, Toronto, ON M5B 2 J1 Canada; Centre for Public Health Nursing, Ballincollig and Bishopstown, Co, Cork, Ireland; Centre for Public Health Nursing, Mahon and Ballintemple, Cork City, Ireland; Health Service Executive of Ireland, South Lee, St Finbarrs Hospital, Douglas Rd, Cork City, Ireland; School of Nursing and Midwifery, University College Cork, Cork, Ireland; COLLAGE (COLLaboration on AGEing), Cork City and Louth Age Friendly County Initiative, Co Louth, University College Cork, Cork, Ireland; Health Research Board, Clinical Research Facility Galway, National University of Ireland, Galway, Ireland

**Keywords:** Screening, Frailty, Risk, Adverse outcomes, Risk Instrument for Screening in the Community (RISC), Clinical Frailty Scale (CFS), and public health nurses (PHNs)

## Abstract

**Background:**

Predicting risk of adverse healthcare outcomes, among community dwelling older adults, is difficult. The Risk Instrument for Screening in the Community (RISC) is a short (2–5 min), global subjective assessment of risk created to identify patients’ 1-year risk of three outcomes:institutionalisation, hospitalisation and death.

**Methods:**

We compared the accuracy and predictive ability of the RISC, scored by Public Health Nurses (PHN), to the Clinical Frailty Scale (CFS) in a prospective cohort study of community dwelling older adults (*n* = 803), in two Irish PHN sectors. The area under the curve (AUC), from receiver operating characteristic curves and binary logistic regression models, with odds ratios (OR), compared the discriminatory characteristics of the RISC and CFS.

**Results:**

Follow-up data were available for 801 patients. The 1-year incidence of institutionalisation, hospitalisation and death were 10.2, 17.7 and 15.6 % respectively. Patients scored maximum-risk (RISC score 3,4 or 5/5) at baseline had a significantly greater rate of institutionalisation (31.3 and 7.1 %, *p* < 0.001), hospitalisation (25.4 and 13.2 %, *p* < 0.001) and death (33.5 and 10.8 %, *p* < 0.001), than those scored minimum-risk (score 1 or 2/5). The RISC had comparable accuracy for 1-year risk of institutionalisation (AUC of 0.70 versus 0.63), hospitalisation (AUC 0.61 versus 0.55), and death (AUC 0.70 versus 0.67), to the CFS. The RISC significantly added to the predictive accuracy of the regression model for institutionalisation (OR 1.43, *p* = 0.01), hospitalisation (OR 1.28, *p* = 0.01), and death (OR 1.58, *p* = 0.001).

**Conclusion:**

Follow-up outcomes matched well with baseline risk. The RISC, a short global subjective assessment, demonstrated satisfactory validity compared with the CFS.

## Background

The number and proportion of older adults in the European Union is growing in the face of limited healthcare resources [1]. This rise is associated with an increased prevalence of functional decline and frailty, in community dwelling older adults [2], creating risk of adverse healthcare outcomes [3]. Identifying those likely to develop adverse outcomes is important, to allocate existing resources more effectively. Rational decision-making in healthcare requires reliable and valid quantitative ways of expressing risk, that balance the potential costs and benefits of different management strategies [4]. While risk assessment, utilizing risk prediction models, is increasing [5], quantitative health impact assessment remains relatively rare in healthcare [4].

The risk of adverse outcomes in community dwelling older adults predisposes to the development of frailty and functional decline [6] and is associated with multiple conditions including depression, cognitive impairment, medical comorbidities and physical inactivity [7–9], factors that can be grouped into three main domains: mental state, activities of daily living (ADL) and medical state. Another important factor is the ability of social networks to manage an individual’s needs [10]. There are several different approaches to identify older adults at risk of adverse outcomes. Some act as rapid screens, others as short surrogates for comprehensive geriatric assessment (CGA) [11]. While many focus on risk of specific outcomes, such as hospital readmission [5], others target frailty [12]. A wide selection of frailty screening instruments are currently available, each with varying psychometric properties [12]. No single approach is regarded as ‘gold-standard’. It is suggested that the combination of a short screen followed by more detailed triage and assessment of high-risk individuals, may be most effective [13]. Although the stratification of risk scores is associated with clinically meaningful gradients for some outcomes [5], most have poor predictive ability [14], particularly at an individual level, possibly reflecting a failure to incorporate important personalised data [5].

In Ireland, Public Health Nurses (PHN) provide the core nursing and midwifery services in the community. They work as part of the primary healthcare multidisciplinary team, with the majority assigned to geographical areas. They take a population health approach to assessment of needs at individual, family and community level [15]. PHNs visit people in their home and are ideally placed to screen, both opportunistically and proactively [16], and deliver medical [17], psychological and social [18] interventions in community settings. In some countries, people with chronic illnesses are more likely to have their condition managed by PHNs than other healthcare professionals [19]. There are high levels of frailty related risk factors in patients under PHN follow-up [20]. Despite this, to our knowledge, no short global risk-screening instrument, designed specifically for community healthcare workers, has been developed. We created the Risk Instrument for Screening in the Community (RISC), to identify those at greatest risk of institutionalisation, hospitalisation and death [21, 22]. The purpose of this study was to determine the accuracy and predictive ability of the RISC, scored by PHNs, to another subjective global assessment and frailty scale, the Clinical Frailty Scale (CFS).

## Methods

### Development of the RISC

The RISC was developed through an iterative process of item generation and reduction, between 2011 and 2012, using literature searches and focus groups with PHNs [21]. The instrument includes demographic data and records the presence (yes or no responses) and magnitude (mild, moderate, severe) of concern across three domains: mental state, ADLs and medical state [22]. Based upon severity of concern and the caregiver networks’ ability to manage them, an overall global subjective assessment of risk score is then assigned to three adverse outcomes: institutionalisation, hospitalisation and death at 1 year from the date of assessment. A simple Likert scale scores five levels of risk from one (minimal and rare) to five (extreme and certain). The RISC is presented in Appendix 1. The RISC instrument, originally called the Community Assessment of Risk Screening Tool or CARST, has excellent inter-rater reliability (Fleiss’ Kappa = 0.86-1.0), internal consistency (Cronbachs’ alpha coefficient = 0.94) and takes 2–5 min to complete [23]. Previous correlation of the RISC domains showed medium to strong correlation with the CFS, Barthel Index (BI), Abbreviated Mental Test Score (AMTS), and Charlson Co-morbidity Index adding to the content validity of the instrument [21].

### Patients

Community dwelling adults over 65 years, currently under follow-up by their PHN, including those living in supervised (sheltered) accommodation. PHNs only review patients after referral, the most common indication for referral being review post hospital discharge [21]. Patients were excluded if they were aged <65 years, currently resident in institutional care (nursing home or other long-term care unit) or no longer under follow-up.

### Data collection

PHN sectors in Cork, Ireland, were approached and invited to participate in the Community Assessment of Risk Tool and Strategies (CARTS) study, an ongoing prospective cohort study of community dwelling older adults, in Southern Ireland. CARTS is part of Irelands three star European Innovation Partnership on Active and Healthy Ageing reference site, COLLAGE (COLLaboration on AGEing) [24–26]. Two PHN sectors covering urban, suburban and some rural areas in Cork, were the first respondents and were subsequently selected by non-probability convenience sampling using a quota method. Given that previous analysis of a risk register suggested that the composite risk of all adverse outcomes in community dwelling older adults in Cork was 7 % [27], it was estimated that for a total population aged >65 years of 4,815 (Central Statistics Office of Ireland, 2006), with a potential margin of error for the confidence interval (CI) estimate of 2 %, that the sample size required to adequately power the study using the hypergeometric distribution formula, $n=\frac{N{z}^2p\left(1-p\right)}{E^2\left(N-1\right)+{z}^zp\left(1-p\right)}$, was 554 patients. Prior to assessing their patients, PHNs (*n* = 15) were trained and certified in scoring the RISC. PHNs only included those directly under their care. Patients were followed for 1 year between March 2012 and August 2013. Additional demographic information was abstracted from the PHN records by a clinician, blinded to the scores [21]. Follow-up data on hospitalisation and death were obtained from the Hospital In-Patient Enquiry department of all hospitals in Cork. Follow-up data on institutionalisation were obtained from the Cork Local Placement Forum which co-ordinates Long Term Care (LTC). General practitioners were also asked to provide additional information on outcomes, in an attempt to identify patient events outside of the catchment area. Although the Clinical Research Ethics Committee of the Cork Teaching Hospitals determined that consent was not required for retrospective chart review, informed written consent or assent where patients were deemed unable to provide consent, was required for all patients included in the CARTS intervention study.

### Outcome measures

Institutionalisation was defined as admission to LTC. In Ireland, LTC encompasses both high (community hospital) and low dependency (general nursing homes) but does not include sheltered accommodation (assisted living /supportive housing programmes), continuing care, retirement communities, or home care. Hospitalisation was defined as an acute admission to an acute (secondary or tertiary referral) hospital, not including elective admissions or planned rehabilitation. Results of standardised testing conducted routinely by PHNs were also recorded including the BI [28] and AMTS [29]. The BI is a 20-point measure of basic ADLs, where a score of 20 indicates independence and zero denotes complete functional dependence. A cut-off of >16 suggests a high functional level. The AMTS is a 10-point score of cognition, where ten suggests normal cognition, zero severe impairment. A cut-off of <7 is suggestive of cognitive impairment. In addition, the PHN scored each patient on the CFS [30]. This validated measure of frailty, is a 9-point scale, scored from one (very fit) to nine (terminally ill) and can be corrected for people with dementia. A score of four is regarded as vulnerable or pre-frail. Scores of ≥5 are regarded as frail and five was used as the optimal cut-off score. Prior to scoring, each PHN stated whether they perceived patients to be frail or not (yes/no). The overall burden of co-morbidity was measured using the Charlson Co-morbidity Index [31].

### Statistical analysis

Data were analyzed with SPSS (20.0). The Shapiro–Wilk test was used to test for normality and found that the majority of data were non-parametric. The Mann Whitney *U* test compared median values. Pearson’s Chi Squared test was used to compare distributions. Correlation with 1-year outcome data was made using either Pearson’s or Spearman (non-parametric) correlation coefficients. Where variables were dichotomous, the point biserial correlation coefficient was calculated. The sensitivity, specificity, positive predictive value (PPV) and negative predictive value (NPV), were also calculated based upon optimal cut-off scores. Accuracy was determined from the area under the curve (AUC), calculated from receiver operating characteristic (ROC) curves. Binary logistic regression models were created using different variables to generate odds ratios (OR), measuring the association between each variable and outcome. Variables initially incorporated into the model included; age, gender, living alone (Yes/No), BI score, number of meds, AMTS score, receiving home help (Yes/No) and the Charlson Co-morbidity Index. This was followed in turn by the CFS and RISC. Kaplan-Meier survival analysis and Cox regression were used to compare time to events for patients scored, at baseline. Patients were divided into minimum-risk (RISC score 1 or 2/5) and maximum-risk (score 3,4 or 5/5) based upon ROC curve analysis.

## Results

The median age of the 803 patients reviewed was 80 years, interquartile range (IQR +/−10). There were 516 females (64 %) and 287 males (36 %). PHNs perception of frailty, without using a standardized frailty instrument, described 335/803 (42 %) patients as frail. The CFS score was available for 784 patients, median score was 5 (+/−2). RISC scores were available for 782 patients (97 %). The majority was scored as minimum-risk (RISC score 1 or 2/5) for the three outcomes of interest (institutionalisation, hospitalisation and death). These are shown in Table 1, which also lists the characteristics of patients according to each outcome. Detailed baseline demographic data were published previously [21].

**Table 1:** Characteristics of patients including Risk Instrument for Screening in the Community (RISC) scores (minimum = score of 1 or 2/5, maximum = 3, 4 or 5/5) according to outcomes: institutionalisation, hospitalisation and death

Characteristic	Institutionalized	Not Institutionalized	P-value	Hospitalized	Not hospitalized	P-value	Dead	Alive	P-value
	*N* = 82 (10.2 %)	*N* = 719 (89.8 %)		*N* = 142 (17.7 %)	*N* = 659 (82.3 %)		*N* = 125 (15.6 %)	*N* = 676 (84.4 %)	
Age (Median ± IQR)	84 ± 10	80 ± 10	0.001	81 ± 10.5	80 ± 10	0.16	82 ± 10	80 ± 10	0.002
Female (%)	63.4 %	64.6 %	0.83	60.6 %	65.3 %	0.28	51.2 %	67.0 %	0.001
Living alone (%)	55.7 %	46.5 %	0.12	47.5 %	47.4 %	0.99	39.8 %	48.9 %	0.07
Cognitive Impairment (%)	58.5 %	25.8 %	<0.001	32.9 %	28.9 %	0.48	36.5 %	28.2 %	0.16
AMTS score (Median ± IQR)	10 ± 3	10 ± 0	<0.001	10 ± 0	10 ± 0	0.65	10 ± 0	10 ± 0	0.49
Barthel Index score (Median ± IQR)	15 ± 7	18 ± 5	<0.001	17 ± 5	18 ± 5	0.03	15 ± 9	18 ± 5	<0.001
Medications (Median ± IQR)	5 ± 4	5 ± 5	0.90	6 ± 5.3	5 ± 5	0.01	7 ± 6	5 ± 4	<0.001
Receiving home help (%)	72.0 %	49.1 %	<0.001	59.9 %	49.6 %	.003	57.6 %	50.3 %	0.13
Hospital length of stay (Median ± IQR)	0 ± 9.8	0 ± 0	<0.001	9.5 ± 15	0 ± 0	<0.001	0 ± 4	0 ± 0	<0.001
Clinical Frailty Scale score (Median ± IQR)	6 ± 1	5 ± 2	<0.001	5 ± 2	5 ± 2	0.03	6 ± 2	5 ± 2	<0.001
PHNs perception of frailty Yes *n* = 335 (42 %)	51.9 %	40.5 %	>0.05	46.0 %	40.8 %	0.26	65.9 %	37.2 %	<0.001
Charlson Comorbidity Index (Median ± IQR)	1 ± 2	1 ± 2	0.48	1 ± 2	1 ± 2	0.08	1 ± 2	1 ± 2	0.56
RISC score for Institutionalisation (*n* = 782)									
Minimum *n* = 686 (88 %)	7.1 %	92.9 %		16.5 %	83.5 %		14.3 %	85.7 %	
Maximum *n* = 96 (12 %)	31.3 %	68.7 %	<0.001	26.0 %	74.0 %	0.02	24.0 %	76.0 %	0.01
RISC score for Hospitalisation (*n* = 782)									
Minimum *n* = 499 (64 %)	7.4 %	92.6 %		13.2 %	86.8 %		9.4 %	90.6 %	
Maximum *n* = 283 (36 %)	14.8 %	85.2 %	0.001	25.4 %	74.6 %	<0.001	26.1 %	73.9 %	<0.001
RISC score for Death (*n* = 782)									
Minimum *n* = 621 (79 %)	8.4 %	91.6 %		16.7 %	83.3 %		10.8 %	89.2 %	
Maximum *n* = 161 (21 %)	16.8 %	83.2 %	0.002	21.1 %	78.9 %	0.20	33.5 %	66.5 %	<0.001

From the total sample assessed, follow-up data were available for 801. Two patients moved location and could not be followed. The incidence of institutionalisation at 1-year was 82/801 (10.2 %). The incidence rate for hospitalisation (at least one) was 142/801 (17.7 %), while the overall mortality rate was 125/801 (5.6 %). Those institutionalised were significantly older, and more cognitively and functionally impaired than those who were not. Age, gender, cognition and social isolation did not influence whether patients were hospitalised. Those alive at 1 year were more likely to be female. At 1-year, there were no significant differences in the burden of co-morbidities between those institutionalised, hospitalised or dead and those who were not. PHNs perception of frailty had a small correlation with risk of death (*r* = 0.21), but not institutionalisation (*r* = 0.07) or hospitalisation, (*r* = 0.04). The CFS only correlated with death, albeit weakly, (*r* = 0.23).

**Fig. 1:**
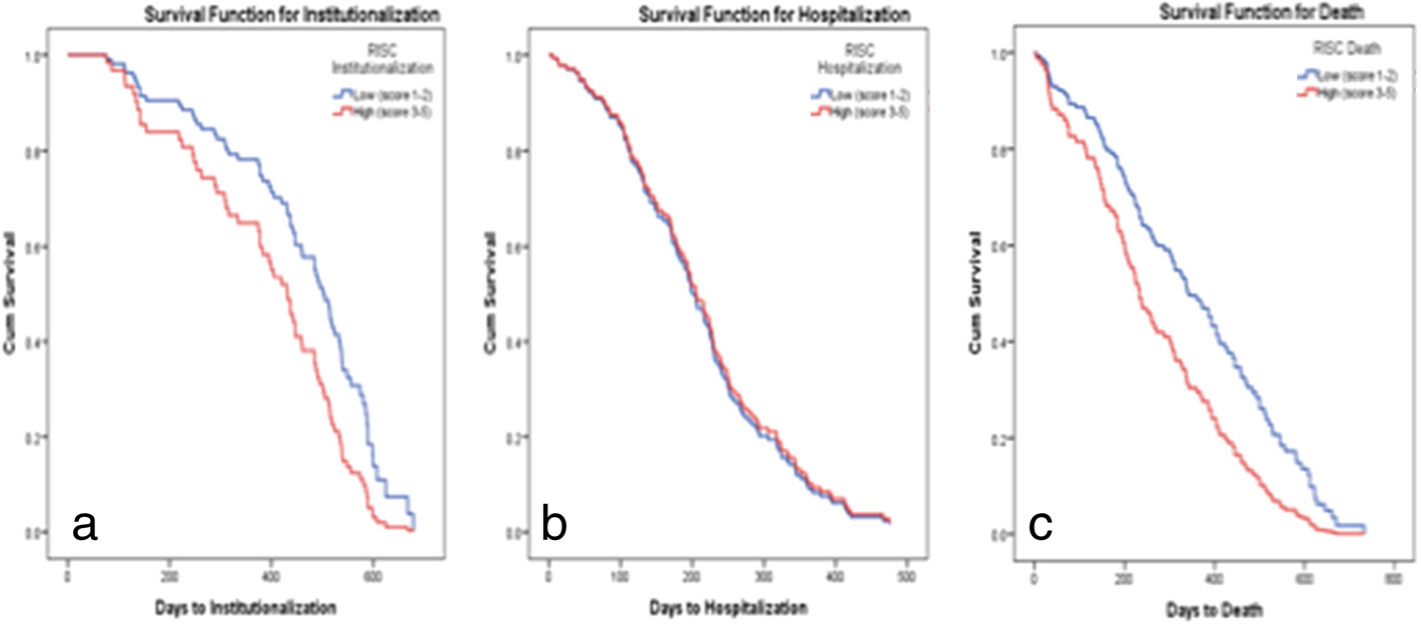
Kaplan-Meier curves representing risk of **a** institutionalisation, **b** hospital admission (at least one) and **c** death, according to the Risk Instrument for Screening in the Community (RISC) classification (minimum versus maximum risk)

Of patients predicted at baseline to be at maximum-risk of institutionalisation, using the RISC, 30/96 (31.3 %) were admitted to LTC in the first year compared to 49/686 (7.1 %) of those scored minimum-risk, *p* < 0.001. Of those scoring maximum-risk for hospitalisation, 72/283 (25.4 %) were admitted versus 66/499 (13.2 %) of minimum-risk patients, *p* < 0.001. The mortality rate of those scoring maximum on the RISC was 54/161 (33.5 %) compared to 67/621 (10.8 %) in the minimum-risk group. Of those scored as frail on the CFS (score of ≥5/9), 60/422 (14.2 %), 85/422 (20.1 %), and 91/422 (21.6 %), were institutionalised, hospitalised or dead at 1 year respectively. When patients deemed pre-frail (CFS score of 4/9) were included, these percentages reduced to 11.8, 19.1 and 17.9 % respectively. Table 2 presents the results of the binary logistic regression models created for each of the three adverse outcomes, assessed with the addition of the CFS in model 2 and the RISC in model 3. The inclusion of the RISC, but not the CFS, significantly increased the predictive power of model one to predict institutionalisation, OR of 1.43 (*p* = 0.01) versus OR of 1.03 (*p* = 0.87) respectively. Similar results were seen for risk of hospitalisation and death. Kaplan-Meier curves (see Fig. 1), adjusted for baseline demographics (age, gender, living alone), compared with Cox regression, showed that the those classified as maximum-risk had a significantly greater time to event than minimum-risk for institutionalisation (OR 1.76, 95 % CI: 1.06–2.94, p = 0.03), and death (OR 1.7, 95 % CI: 1.15–2.52, *p* = 0.009), but not hospitalisation (OR 0.95, 95 % CI: 0.67–1.36, *p* = 0.79).

**Table 2:** Binary logistic regression models comparing predictors of (a) institutionalization, (b) hospitalisation and (c) death. Models 2 (a, b, c) include the Clinical Frailty Scale, model 3 (a, b, c) the RISC score, with the significance value of *model change* denoting increased predictive accuracy of the model relative to models 1 (a, b, c) respectively

(a) Predictors of Institutionalisation	Model 1a	Model 2a	Model 3a
	(95 % CI)	(95 % CI)	(95 % CI)
		(*model change p* = 0.87)	(*model change p* = 0.01)
Age	1.05* (1.01–1.09)	1.05* (1.01–1.09)	1.04 (1.00–1.09)
Gender (Male)	1.31 (0.73–2.33)	1.31 (0.73–2.34)	1.33 (0.74–2.38)
Living Alone	1.69 (0.92–3.10)	1.70 (0.92–3.13)	1.62 (0.88–3.00)
Barthel Index score	0.94 (0.88–1.01)	0.95 (0.87–1.03)	0.96 (0.88–1.05)
Total number of medications	0.97 (0.89–1.06)	0.97 (0.89–1.06)	0.96 (0.88–1.05)
AMTS score	0.84* (0.74–0.95)	0.84* (0.75–0.95)	0.88* (0.77–0.99)
Receiving home help	1.80 (0.95–3.43)	1.79 (0.94–3.42)	1.70 (0.88–3.28)
Charlson Comorbidity Index	1.00 (0.81–1.22)	0.99 (0.81–1.22)	0.96 (0.78–1.19)
Clinical Frailty Scale		1.03 (0.76–1.38)	
RISC score for Institutionalisation			1.43* (1.09–1.88)
**(b) Predictors of Hospitalisation**	Model 1b	Model 2b	Model 3b
	(95 % CI)	(95 % CI)	(95 % CI)
		(*model change p* = 0.65)	(*model change p* = 0.01)
Age	1.02 (0.99–1.05)	1.02 (0.99–1.05)	1.02 (0.99–1.05)
Gender (Male)	1.12 (0.72–1.73)	1.12 (0.72–1.74)	1.13 (0.73–1.76)
Living Alone	1.22 (0.78–1.91)	1.24 (0.79–1.94)	1.23 (0.78–1.92)
Barthel Index score	0.98 (0.93–1.04)	0.99 (0.93–1.06)	1.00 (0.95–1.06)
Total number of medications	1.05 (0.98–1.11)	1.05 (0.98–1.11)	1.03 (0.97–1.10)
AMTS score	1.01 (0.90–1.13)	1.01 (0.90–1.13)	1.00 (0.90–1.13)
Receiving home help	1.39 (0.88–2.19)	1.37 (0.86–2.17)	1.36 (0.86–2.15)
Charlson Comorbidity Index	1.17* (1.02–1.34)	1.16* (1.01–1.33)	1.10 (0.95–1.27)
Clinical Frailty Scale		1.05 (0.85–1.30)	
RISC score for Hospitalisation			1.28* (1.06–1.54)
**(c) Predictors of Death**	Model 1c	Model 2c	Model 3
	(95 % CI)	(95 % CI)	(95 % CI)
		(*model* change *p* = 0.19)	(*model* change *p* = 0.001)
Age	1.05* (1.02–1.09)	1.05* (1.02–1.09)	1.04* (1.01–1.08)
Gender (Male)	1.76* (1.07–2.89)	1.82* (1.10–2.99)	1.87* (1.13–3.10)
Living Alone	1.36 (0.80–2.32)	1.44 (0.84–2.46)	1.23 (0.72–2.11)
Barthel Index score	0.88* (0.83–0.93)	0.91* (0.84–0.98)	0.90* (0.84–0.95)
Total number of medications	1.06 (0.99–1.14)	1.06 (0.99–1.13)	1.06 (0.99–1.14)
AMTS score	1.10 (0.97–1.25)	1.10 (0.97–1.25)	1.11 (0.97–1.26)
Receiving home help	0.75 (0.44–1.29)	0.73 (0.42–1.25)	0.84 (0.49–1.46)
Charlson Comorbidity Index	1.54* (1.32–1.79)	1.51* (1.29–1.76)	1.34* (1.13–1.60)
Clinical Frailty Scale		1.18 (0.92–1.51)	
RISC score for Death			1.58* (1.20–2.08)

Model 1a: *R* ^*2*^ 6.0–12.4 %, *χ* ^*2*^(8) = 38.89, *p* < 0.001; Model 2a: *R* ^2^ 7.1–13.5 %, *χ* ^2^(9) = 38.92, *p* < 0.001; Model 3a: *R* ^2^ 7.0–14.4 %, *χ* ^2^(9) = 45.37, *p* < 0.001

Model 1b: *R* ^*2*^ 2.7–4.4 %, *χ* ^2^(8) = 17.23, *p* = 0.03; Model 2b: *R* ^2^ 2.8–4.4 %, *χ* ^2^(9) = 17.44, *p* = 0.04; Model 3b: *R* ^*2*^ 3.7–6.0 %, *χ* ^2^(9) = 23.88, *p* < 0.01

Model 1c: *R* ^*2*^ 13.8–23.6 %, *χ* ^2^(8) = 92.76, *p* < 0.001; Model 2c: *R* ^*2*^ 14.0–24.0 %, *χ* ^*2*^(9) = 94.46, *p* < 0.001; Model 3c: *R* ^*2*^ 14.8–25.5 %, *χ* ^*2*^(9) = 100.42, *p* < 0.001

Pseudo R-square reported as Cox & Snell - Nagelkerke values, each predictor is reported are odds ratios and * indicates statistically significant values (p < 0.05)

**Fig. 2:**
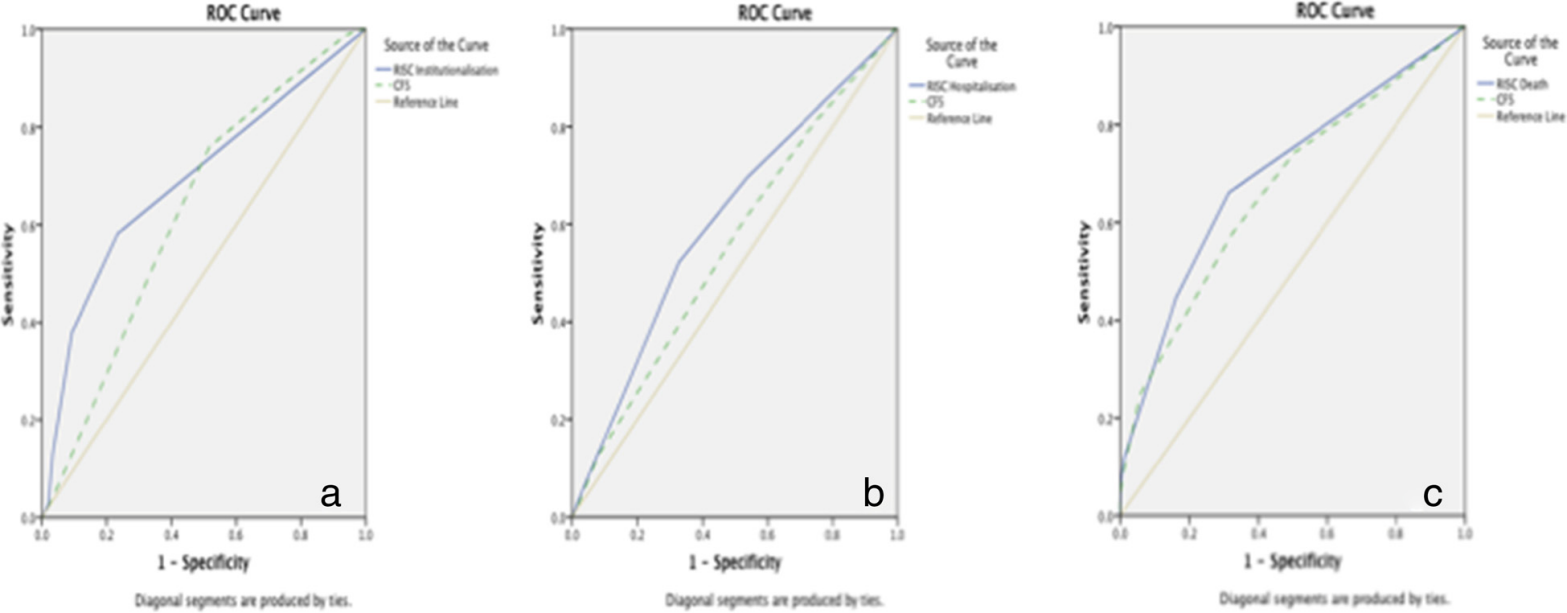
Receiver operating characteristic curve demonstrating sensitivities and specificities of the Risk Instrument for Screening in the Community (RISC) and Clinical Frailty Scale (CFS) in identifying 1-year risk of **a** institutionalisation, **b** hospital admission (at least one) and **c** death

Figure 2 shows ROC curves demonstrating the accuracy of the RISC and the CFS in predicting each outcome. The RISC and CFS had comparable accuracy at predicting institutionalisation (AUC of 0.70 [95 % CI: 0.62–0.76] versus 0.63 [95 % CI: 0.57–0.67] respectively), hospitalisation (AUC of 0.61 [95 % CI: 0.55–0.66] versus 0.55 [95 % CI: 0.50–0.61]), and death (AUC of 0.70 [95 % CI: 0.64–0.75] versus 0.67 [95 % CI: 0.61–0.72]). These differences did not reach statistical significance. Table 3 compares the sensitivity, specificity, PPV and NPV for the CFS, RISC and the PHN perception of frailty. The predictive validity of both instruments was compared using an optimal cut-off score of ≥2 for the RISC and ≥5 for the CFS, based upon the sensitivity and specificity, calculated a priori, from the ROC curves. This cut-off for the CFS is the same as the established frailty cut-off [30]. The CFS had higher sensitivity for most of the three outcomes but lower specificity. The RISC was most sensitive for hospitalization (70 %), the CFS most sensitive for institutionalization (76 %). The PHNs’ perception of frailty had lower sensitivity and specificity for most of the three outcomes. Increasing the cut-off score for the RISC to ≥3, corresponding to the cut-off for maximum-risk, increased the specificity of the RISC, but reduced its sensitivity.

**Table 3:** Comparison of the sensitivity, specificity, positive predictive value (PPV) and negative predictive value (NPV) including 95 % confidence intervals (CI) for each outcome between the public health nurses’ (PHNs) perception of frailty, the Clinical Frailty Scale (CFS) and the Risk Instrument for Screening in the Community (RISC), taking a cut-off of ≥2 (based on ROC curve analysis)

Outcome	Variable	Sensitivity	Specificity	PPV	NPV
		(95 % CI)	(95 % CI)	(95 % CI)	(95 % CI)
Institutionalization	PHNs perception of frailty	52 %	59 %	13 %	92 %
		(48–55 %)	(56–63 %)	(9–16 %)	(89–94 %)
	CFS	76 %	48 %	14 %	95 %
		(73–79 %)	(45–52 %)	(11–18 %)	(92–97 %)
	RISC	58 %	76 %	22 %	94 %
		(55–62 %)	(73–79 %)	(16–27 %)	(92–96 %)
Hospitalization	PHNs perception of frailty	46 %	59 %	19 %	84 %
		(42–49 %)	(56–63 %)	(15–24 %)	(80–87 %)
	CFS	61 %	47 %	20 %	85 %
		(58–65 %)	(44–51 %)	(16–24 %)	(81–89 %)
	RISC	70 %	46 %	22 %	88 %
		(66–73 %)	(43–50 %)	(18–26 %)	(84–91 %)
Death	PHNs perception of frailty	66 %	63 %	25 %	91 %
		(63–69 %)	(59–66 %)	(20–30 %)	(88–93 %)
	CFS	74 %	50 %	22 %	91 %
		(71–77 %)	(46–53 %)	(18–25 %)	(88–94 %)
	RISC	66 %	69 %	28 %	92 %
		(63–69 %)	(65–72 %)	(23–33 %)	(89–94 %)

Comparing the predictive ability of global RISC scores, for other outcomes apart from their indications, found that a significantly larger percentage of patients classified as maximum-risk on the global RISC score for one outcome (e.g. institutionalisation) also experienced another outcome (e.g. hospitalised or dead at 1 year), compared to those classified as minimum-risk, see Table 1. The RISC score for institutionalisation was significantly more accurate at identifying institutionalisation than hospitalisation (*p* < 0.001) or death (*p* = 0.03). The RISC score for hospitalisation was significantly more accurate at predicting death (AUC of 0.70, *p* = 0.03), while the RISC score for death was significantly more accurate at identifying death than institutionalization (*p* = 0.05) and hospitalization (*p* < 0.001).

## Discussion

This study compares the ability of a short global subjective assessment of risk, the RISC, to identify adverse outcomes with the CFS, a validated measure of frailty, and PHNs perception of frailty in a sample of community dwelling older adults in Cork, Ireland. In this sample there was a high incidence of all three adverse outcomes, reflective of the frail nature of patients under PHN follow-up. While there were some significant differences between those institutionalised, hospitalised or dead, at one year, and those who were not, traditional markers of risk including age, gender, and living alone, were inconsistently associated with 1-year outcomes. Regression modeling showed that the RISC increased the predictive accuracy of a model that included common patient variables and assessment scores, while the addition of the CFS had no significant effect. The results suggest that the RISC had comparable accuracy to the CFS in predicting institutionalisation, hospitalisation and death. Although AUC scores suggested that both tests were relatively poor at differentiating patients and differences were not statistically significant, the RISC identified those at greatest risk of all three adverse healthcare outcomes in a clinically meaningful way. Patients at maximum-risk were approximately four times more likely to be institutionalised, twice as likely to be hospitalised and three times more likely to die at 1 year follow-up than those in the minimum-risk category. The differences were all statistically significant. Similarly, those classified as maximum-risk had a significantly shorter median time to institutionalisation and death. While the global RISC scores correctly classified more patients as maximum-risk for each corresponding outcome, there was crossover, suggesting that a global subjective assessment of risk is a general marker for increasing susceptibility to all adverse events. Only the RISC score for death was unable to significantly separate individuals at minimum and maximum risk of hospitalisation. Indeed, the RISC score for death was significantly more accurate in identifying risk of hospitalisation than the RISC score for hospitalisation. This supports data suggesting that hospital admission is difficult to predict and most instruments have poor accuracy in identifying hospitalization and readmission [5].

The CFS was less useful in stratifying patients according to all three outcomes. Most frailty instruments are designed exclusively to predict frailty. The CFS focuses primarily on activity levels and ability to perform ADLs. The RISC incorporates mental state, ADLs and medical problems, in the context of the caregiver network. In this respect, it is a holistic measure, incorporating more domains and contextualising problems to create an individualised measure of risk. Given that frailty is a state of increased vulnerability [32], the RISC may act as a surrogate measure of this vulnerability, operationalising frailty as a risk of three important adverse outcomes: death, hospitalisation and institutionalisation within the context of the caregiver networks’ ability to manage a patients’ care. Furthermore the CFS, because of its lower specificity, <50 % for each of the three outcomes, was less efficient in identifying and triaging older adults. In this study 22 % of patients were deemed pre-frail and 54 % frail, using the CFS, resulting in the need to triage larger numbers for further comprehensive assessment and management. A much smaller number of patients were identified as maximum-risk using the RISC [21]. In addition, the PHNs perception of frailty, albeit a crude measure of frailty (frail, yes or no), performed well compared to both the RISC and CFS, suggesting that simple qualitative judgements made by healthcare professionals, with detailed knowledge of their population, may be sufficient. Simple subjective assessments have been used successfully in other studies. For example the “surprise” question is an independent predictor of 1-year mortality [33] and is validated used in different clinical settings [34, 35].

This paper has several limitations. The data collection was based upon a retrospective review of PHN records, some of which were incomplete. The retrospective nature of the chart review also limited the variables that could be included in this analysis such that instruments like the AMTS [36] and Charlson Co-morbidity Index [37] are criticized for their poor accuracy. Like all screening instruments they suggest the need for further assessment rather than a specific diagnosis. These instruments and others such as the BI are continuous variables that may have affected the regression analysis. However, each continuous variable was explored and a somewhat linear association was found with each of the adverse healthcare outcomes and there is no accepted way to categorise these variables in community samples. Further study should explore the transformation of such variables into categorical data using statistical techniques such as cluster analysis. This could also provide a stratification of risk according to age and functional levels. The prevalence of frailty was high, which affects the ability to interpret the PPV and NPV analysis. The method of sampling may also have led to a large degree of selection bias in that patients under PHN follow-up are at higher risk of adverse outcomes. The study was conducted by patient’s PHN, each of whom were trained before scoring the RISC. However, the reliability and validity of the CFS, scored by PHNs was not examined, which may have led to bias. In addition, none of the measures used are considered ‘gold-standard’. Inclusion of objective observer-rated instruments [38, 39] could have reduced potential bias relating to the PHNs perception of frailty. The strengths of this paper include the comprehensive nature of the PHN records, PHNs knowledge of their patients and the inclusion of a large cross-sectional and representative community sample of patients under PHN follow-up. The RISC is currently being validated in several other countries including Australia [40], Northern Ireland, Portugal and Spain. Given the paucity of risk prediction instruments in community settings [41], future studies should compare the RISC and its subtests [42] to comprehensive instruments such as the InterRAI home assessment [43]. Studies should also investigate if assessments like the RISC, scored by healthcare providers working in the community, could be used to triage patients deemed most at risk and pre-frail-frail for CGA.

## Conclusions

In summary, the RISC predicted adverse outcomes in a community cohort of older adults, such that those at maximum-risk were significantly more likely to be institutionalizsed, hospitalised or die at follow-up, than those at minimum-risk. The RISC, a short (2–5 min) risk prediction screen, was better than a more traditional frailty scale (the CFS) in predicting outcomes. This study suggests the potential of a short global subjective risk assessment, scored by trained healthcare workers, familiar with their patients, as an alternative to short frailty measures in the prediction of adverse healthcare outcomes. Given the limitations of the study and potential for bias, further research is needed to confirm the external validity of the RISC in different settings and to compare it with more detailed assessment instruments.
